# Effect of glycosylation on protein folding: From biological roles to chemical protein synthesis

**DOI:** 10.1016/j.isci.2025.112605

**Published:** 2025-05-08

**Authors:** Chunchun Hao, Qijie Zou, Xuerong Bai, Weiwei Shi

**Affiliations:** 1 Ministry of Education Key Laboratory of Radiopharmaceuticals, College of Chemistry, Beijing Normal University, Beijing 100875, China; 2 Ministry of Education Key Laboratory of Bioorganic Phosphorus Chemistry and Chemical Biology, Department of Chemistry, Tsinghua University, Beijing 100084, China

**Keywords:** Biological sciences, Chemistry, Protein

## Abstract

Chemical protein synthesis has become an important tool in biotechnology and synthetic biology for producing proteins with complex structures. However, achieving correct folding *in vitro* remains a significant challenge. Glycosylation, a ubiquitous modification, stabilizes folding intermediates, prevents aggregation, and accelerates folding in both cellular and cell-free systems. In this review, we discuss the dual role of glycosylation in biological systems and *in vitro* experiments, focusing on how it promotes protein folding and stability. We also discuss the temporary glycosylation scaffold strategy for chemical protein synthesis, which offers a reversible approach to guide protein folding without leaving permanent modifications. This strategy provides a promising solution to the challenges of *in vitro* folding and holds significant potential to produce therapeutic proteins and the development of synthetic proteins with precise structural requirements.

## Graphical abstract

**Figure:**
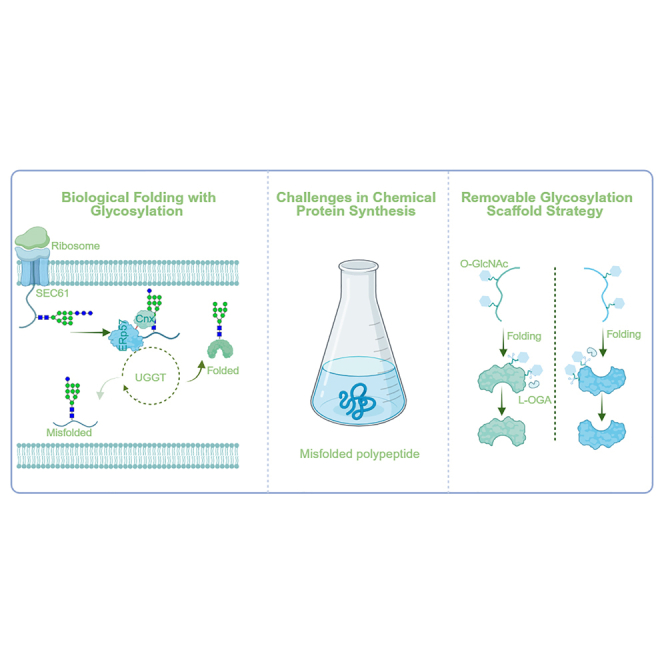

## Introduction

The ability to produce proteins through chemical synthesis and biological recombinant has significantly advanced the fields of biotechnology,^1^^,^^2^^,^^3^^,^^4^^,^^5^ pharmaceuticals,^6^^,^^7^^,^^8^^,^^9^^,^^10^^,^^11^ and synthetic biology.^12^^,^^13^^,^^14^^,^^15^^,^^16^^,^^17^^,^^18^^,^^19^ While biological recombinant techniques utilize the cellular machinery of organisms for protein production, chemical synthesis (based on native chemical ligation and advanced desulfurization strategies^20^^,^^21^) provides a flexible alternative for creating proteins with non-natural amino acids or specific structural features that are difficult to produce biologically.^22^^,^^23^^,^^24^^,^^25^^,^^26^^,^^27^^,^^28^^,^^29^^,^^30^ However, one of the significant challenges in chemical synthesis is achieving correct protein folding *in vitro*, particularly in the absence of cellular mechanisms such as glycosylation.^31^^,^^32^^,^^33^^,^^34^^,^^35^

Glycosylation, a prevalent post-translational modification (PTM) across eukaryotic systems, plays a pivotal role in protein folding, stability, and functionality.^36^^,^^37^^,^^38^ This review specifically focuses on the role of glycosylation in biotechnology applications, emphasizing its utility in enhancing the solubility, stability, and folding of proteins during biomanufacturing. The process is predominantly discussed within the context of eukaryotic glycoproteins, encompassing yeast and mammalian systems, which represent the most commonly utilized models for studying glycosylation dynamics and applications in protein synthesis.

Chemically synthesized proteins produced outside the cellular environment face substantial challenges due to the absence of natural folding pathways and PTMs such as glycosylation.^39^^,^^40^^,^^41^^,^^42^ Recent advances have sought to harness glycosylation as a strategic tool in this context.^32^^,^^43^^,^^44^^,^^45^^,^^46^ Unlike its permanent role in natural systems, we propose using glycosylation as a temporary scaffold—a transient modification that supports structural integrity during folding but is removed once the protein achieves its correct conformation. This innovative approach mimics the stabilizing effects of glycosylation seen in biological systems, enabling precise control over the folding environment and addressing the inherent challenges of *in vitro* protein synthesis.^33^^,^^47^^,^^48^ This review elucidates the role of glycosylation in biological systems, underscores the challenges of folding chemically synthesized proteins, and introduces our novel glycosylation strategy as a removable scaffold that facilitates correct folding. Through a focused discussion on glycosylation’s role in biotechnology, particularly in chemical protein synthesis, we aim to provide insights into its application as both a fundamental biological process and a biotechnological tool.

## The role of glycosylation in Co-translational and post-translational protein folding

Glycosylation is integral to protein maturation, particularly in facilitating proper protein folding. The folding process is highly complex and tightly regulated, requiring the coordinated interplay of various organelles and molecular chaperones.^49^

### Co-translational protein folding on the ribosome

During co-translational translocation into the ER, nascent polypeptides receive initial glycosylation modifications via oligosaccharyltransferase (OST), which contributes to early folding events and stabilization prior to complete synthesis.^50^,^51^ During co-translational translocation, the signal recognition particle (SRP) recognizes the N-terminal signal peptide of the nascent protein, temporarily pausing synthesis while the ribosome-protein complex is transferred to the SRP receptor on the ER membrane.^52^ The nascent protein is then inserted into the translocon, a membrane-bound protein-conducting channel composed of the secretory 61 (Sec61) translocation complex in eukaryotes. Once inside the ER, the signal sequence is cleaved from the core protein by signal peptidase, and the protein undergoes proper folding and post-translational modifications. This pathway is utilized by secretory proteins, membrane-bound proteins, and proteins destined for the ER, Golgi apparatus, or endosomes.^53^ In particular, approximately one-third of all proteins in eukaryotic cells are translocated into the ER for proper folding, post-translational modifications, and trafficking. These include secretory proteins (e.g., insulin, antibodies), integral membrane proteins (e.g., EGFR, transporters), and lysosomal enzymes (e.g., cathepsins). In contrast, cytoplasmic proteins, such as glycolytic enzymes and structural proteins, are synthesized on free ribosomes and do not enter the ER.

### Protein folding and quality control in the endoplasmic reticulum

**Figure 1:**
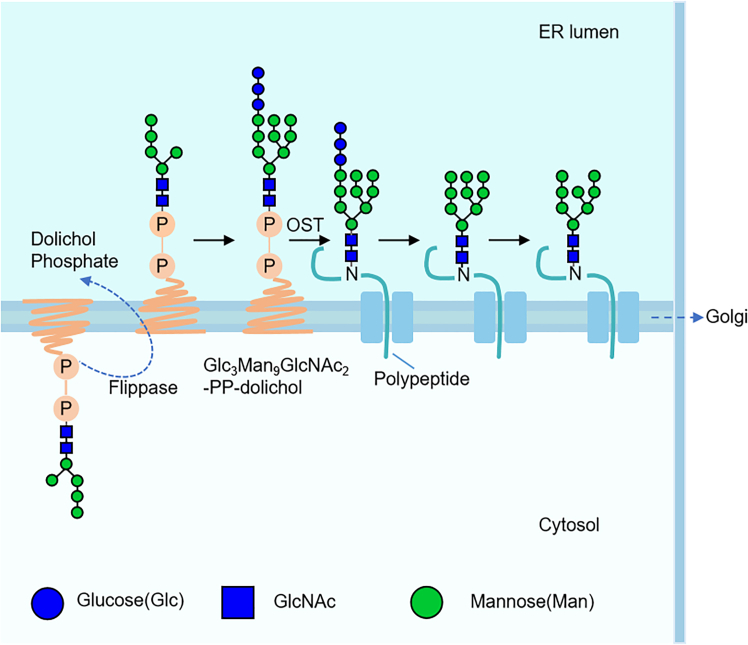
The initial formation process of N-glycosylation in the ER N-glycosylation is initiated in the endoplasmic reticulum, where a core glycan is transferred to the nascent peptide chain by glycosyltransferase, and then transported to the Golgi apparatus for further processing.

N-glycosylation is initiated in the endoplasmic reticulum (ER) with the transfer of GlcNAc2Man5 to dolichol phosphate, followed by translocation via a flippase. Additional residues are then added to form Glc3Man9GlcNAc2-PP-dolichol. This glycan is enzymatically transferred to the nascent peptide chain by oligosaccharyltransferase (OST), which is embedded in the ER membrane. OST transfers the core glycan group (Glc3Man9GlcNAc2) from dolichol to a specific Asn residue within the consensus sequence Asn-X-Ser/Thr of the nascent polypeptide, where X represents any amino acid except proline. Afterward, the glycopeptide exits the ER and is transported to the Golgi apparatus for further processing, including glycan trimming and branching (Figure 1).^54^

#### N-glycosylation and its role in protein folding in the endoplasmic reticulum

##### Thermodynamic contributions of N-glycosylation to protein folding

N-glycosylation influences protein folding directly through the structural attributes of the glycans attached. Typically, these glycans are complex, branched structures such as complex or hybrid types, which can significantly influence the physical properties of the protein. The addition of such glycans increases the molecular rigidity and stability of the protein, thereby facilitating proper folding and enhancing stability against environmental stresses. Waespy et al. demonstrated how complex N-glycans on recombinant proteins directly enhance their stability and folding by structurally “locking” certain protein domains in the correct orientation, which is crucial for functional activity.^55^

Conversely, glycosylation also plays a crucial role in the recruitment of molecular chaperones, predominantly involving high-mannose or paucimannose structures. These simpler glycans are recognized by chaperones such as calnexin and calreticulin within the ER, initiating a quality control mechanism that assists in the proper folding of the glycoprotein. The function here is more about ensuring fidelity in folding rather than enhancing structural stability directly.^56^

The primary structural distinction lies in the complexity and type of glycan involved. Complex and hybrid glycans typically engage directly in stabilizing protein conformations and enhancing folding kinetics due to their extensive branching and large size, which impacts the protein’s physical landscape. In contrast, simpler high-mannose glycans are more involved in signaling and recognition processes, crucial for chaperone-mediated quality control. These differences underscore distinct roles: structural modification versus functional interaction with cellular machinery.

Understanding these structural distinctions and their functional implications provides crucial insights into how glycosylation can be harnessed in biotechnology and therapeutic protein production. By distinguishing between the types of glycans involved in direct folding effects and those essential for chaperone recruitment, researchers can better design glycoproteins with optimized stability and functionality.^57^^,^^58^

##### Endoplasmic reticulum chaperone-mediated quality control of glycoproteins

**Figure 2:**
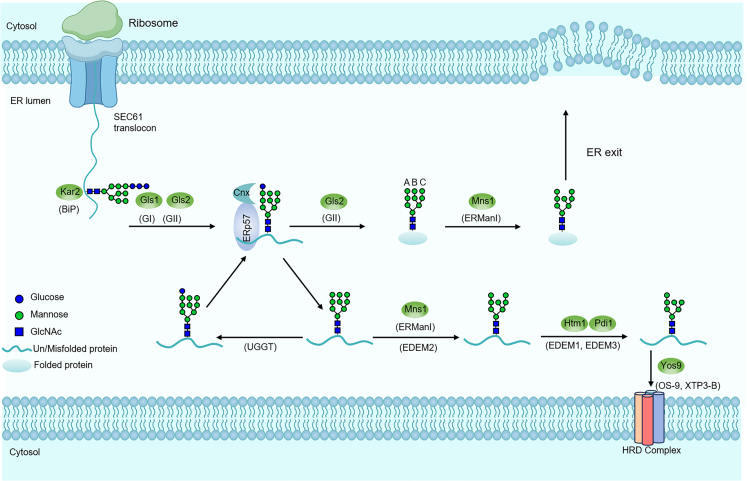
ER-associated degradation process of N-glycosylation After adding the pre-assembled oligosaccharide, the two outermost glucose residues are removed, allowing the nascent polypeptide to associate with Cnx and ERp57. Most glycopolypeptides are released as native proteins and exit the ER, while folding-defective polypeptides enter cycles of dissociation and reassociation with Cnx. Eventually, extensive de-mannosylation halts folding attempts, leading to the retrotranslocation of misfolded polypeptides into the cytosol for degradation. Yeast proteins are shown as green ovals, with their mammalian homologues listed later in discussion.

N-glycan-mediated protein folding and quality control is a highly intricate and tightly regulated process, predominantly taking place in the ER (Figure 2).^37^ The distinct structure and composition of N-glycan chains can serve as “encoded signals” that direct protein folding by interacting with molecular chaperones, including calnexin and calreticulin.

The sequential trimming of glycan chains, such as the removal of glucose residues, alters the glycan’s structure, modulating its binding strength and specificity with chaperone proteins and thus regulating the protein folding process. For example, glucosidases I and II (GⅠ, GⅡ) trim the core oligosaccharide by removing two glucose residues, yielding the Glc1Man9GlcNAc2 oligosaccharide. This intermediate is transiently recognized by lectin-like chaperones, including calnexin (Cnx) and its soluble homolog, calreticulin (Crt).^59^ Following this interaction, disulfide bonds are introduced into the unfolded protein substrate via the assistant action of protein disulfide isomerases (PDI), such as ERp57.^37^

When the protein fails to fold correctly, specific structures on the N-glycan chain are recognized by UDP-glucose: glycoprotein glucosyltransferase (UGGT), which reattaches glucose residues to the glycan, allowing the protein to rebind to chaperones and undergo another folding cycle. When the protein cannot be brought to its correct state through refolding mechanisms, structural alterations in the N-glycan lead to its recognition by specific receptors, such as Osteosarcoma-9 (OS-9) and XTP3-transactivated Protein B (XTP3-B). These receptors direct misfolded proteins toward the endoplasmic reticulum-associated degradation (ERAD) pathway, ensuring the degradation of misfolded proteins, preventing their intracellular accumulation, which could otherwise lead to pathological conditions.^60^^,^^61^

Protein quality control is governed by a multitude of factors and has been extensively investigated in both *Saccharomyces cerevisiae* and mammalian tissue culture systems. Despite similarities, notable differences in the mechanisms of ERAD exist between these two systems.

In *Saccharomyces cerevisiae*, the trimming of glucosyl residues in the ER is a crucial step for initiating glycan-dependent ERAD. In the ER, nascent glycoproteins undergo N-glycosylation, initially acquiring a Glc3Man9GlcNAc2 structure. Sequential trimming of glucose residues by glucosidases I and II generates monoglucosylated glycans, facilitating interaction with calnexin and calreticulin to assist in proper folding. Misfolded glycoproteins are further processed by ER mannosidase I (Mns1) and Hsp70-type molecular chaperone 1/ mannose-like protein 1 (Htm1p/EDEM1), which trim mannose residues to produce a Man8GlcNAc2 glycan. This specific structure is recognized by lectins such as Yos9p in yeast or OS-9 in mammals, marking the glycoprotein for degradation via the ERAD pathway. Mns1 further process the glycan chain by removing the terminal α-1,2-linked mannose residue on the B-branch (a branch of the oligomannose in Figure 2), producing the intermediate Man8GlcNAc2. At this stage, the protein is recognized as an ERAD substrate.^62^ The substrate is then translocated to ER membrane channels, such as the SEC61 translocon, with potential assistance from chaperone proteins, such as Hypermannosylation 1 (Htm1), to facilitate its transport into the cytosol.^63^ Once in the cytosol, the ERAD substrate undergoes ubiquitination, and the ubiquitinated protein is subsequently directed by cytosolic chaperones to the proteasome for degradation.^64^

The ERAD mechanism in mammals is more complex than that in *Saccharomyces cerevisiae*, involving a greater number of regulatory factors. ER degradation enhancing EDEM1 and ER degradation enhancing mannose-like protein 3 (EDEM3) are key enzymes involved in ERAD in mammals, and they trigger ERAD by converting the Man9GlcNAc2 glycan chain into Man7GlcNAc2 through a two-step process.^65^ EDEM1 collaborates with redox chaperones, including Endoplasmic Reticulum DnaJ Homolog 5 (ERdj5/DNAJC10), to reduce disulfide bonds in misfolded proteins, facilitating their preparation for ERAD.^66^ Once de-glycosylated and reduced, misfolded proteins are transported to the cytosol via the ERAD pathway, where they are ubiquitinated and ultimately degraded by the proteasome.^67^

#### O-mannosylation and protein folding in the endoplasmic reticulum

**Figure 3:**
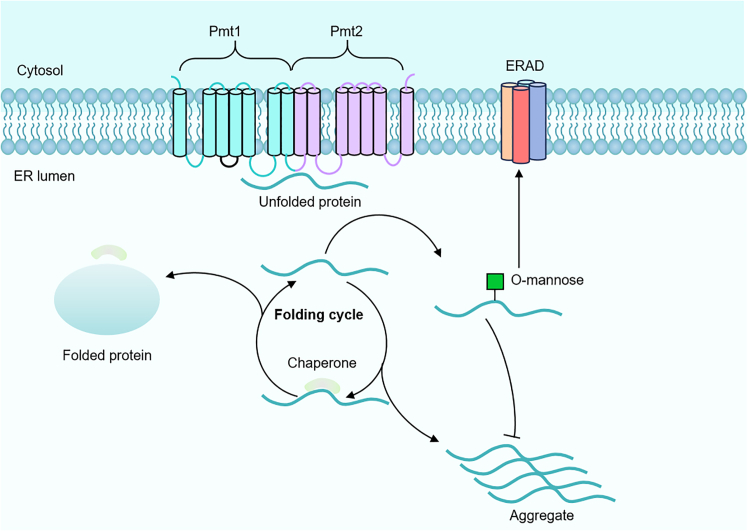
ER-associated degradation process of O-mannose glycosylation A newly synthesized unfolded protein interacts with a chaperone to initiate folding. It can either fold successfully or return for another attempt. If it fails to fold within a specific time frame, it is targeted for O-mannosylation by the Pmt1–Pmt2 complex, which removes it from the folding cycle, prevents aggregation, and facilitates its entry into ERAD.

In *Saccharomyces cerevisiae*, O-glycosylation, particularly O-mannosylation, plays a crucial role in facilitating protein folding and maintaining quality control (Figure 3). O-mannosylation is mediated by O-glycosyltransferases, with the protein O-mannosyltransferase 1-protein O-mannosyltransferase 2 (Pmt1-Pmt2) complex being the primary enzyme responsible for the addition of mannose to Ser/Thr residues in proteins.^68^ These enzymes are integral to the protein folding quality control process. O-mannosylation helps terminate inefficient folding cycles within the ER, preventing misfolded proteins from re-entering the folding process, inhibiting aggregation, and directing them toward the ERAD pathway. Additionally, O-mannosylation is thought to regulate folding efficiency by modifying the intrinsic folding capacity of proteins. It may also affect protein interactions with ER chaperones (such as karyoplasmic 2/binding immunoglobulin protein), potentially terminating ineffective early folding attempts by reducing chaperone binding.^69^^,^^70^

### Glycosylation processing and function in the Golgi

While the initial transfer of sugars to glycoproteins occurs in the ER or on the ER membrane, the subsequent addition of various sugars that form mature glycans is completed in the Golgi apparatus.^38^ In the Golgi apparatus, glycan processing is a highly dynamic and complex process, involving the coordinated action of multiple glycosyltransferases (GTs) and other glycosylation-related enzymes. The Golgi apparatus is organized into distinct compartments, each responsible for specific glycosylation reactions. Proteins shuttle between these compartments, gradually completing the complex glycosylation modifications before being accurately sorted and packaged into vesicles. These glycosylated proteins are then transported via vesicles through the intracellular trafficking network to their final destinations, such as the cell membrane, lysosomes, or other organelles.^71^ Given the complexity of glycan processing within the Golgi apparatus, we will introduce several cases to highlight the importance of Golgi-mediated glycan processing for protein maturation.

#### N-glycan processing and functional modifications in the Golgi

**Figure 4:**
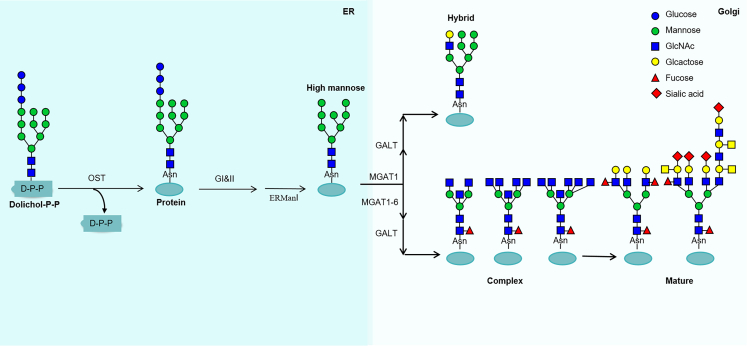
N-glycan processing in the Golgi apparatus In the endoplasmic reticulum, after glycan trimming and quality control, the N-glycan takes on a high-mannose form. This glycan is further processed in the Golgi apparatus, leading to the formation of the hybrid and complex glycans. Most hybrid and complex N-glycans are subsequently modified by the elongation of N-acetylglucosamine (GlcNAc) residues with the addition of other sugars and subsequent capping with fucose, sialic acid, galactose, or GlcNAc.

The mannose-6-phosphate (M6P) pathway is a critical mechanism for targeting lysosomal enzymes to lysosomes. In this pathway, lysosomal enzymes synthesized in the rough endoplasmic reticulum are tagged with M6P residues in the Golgi apparatus. This tagging involves the addition of GlcNAc-1-phosphate to mannose residues, followed by the removal of GlcNAc to expose the M6P marker.^72^ Mannose-6-phosphate receptors (MPRs) in the *trans*-Golgi network recognize these markers, facilitating the transport of the enzyme-receptor complexes to endosomes.^73^ In the acidic environment of endosomes, the enzymes dissociate from the receptors and are subsequently delivered to lysosomes, where they become active in degrading various biomolecules. Disruptions in the M6P pathway can lead to the misrouting of lysosomal enzymes, resulting in lysosomal storage disorders.^74^ Notably, glycosylation within the Golgi apparatus plays a pivotal role in the transport of lysosomal enzymes, independent of the mannose-6-phosphate M6P pathway (Figure 4). N-linked glycoproteins transported from the ER to the Golgi initially carry a Man8GlcNAc2 glycan chain. In the Golgi, N-glycans undergo partial mannose removal by α-mannosidase, generating the Man5GlcNAc2Asn intermediate. In the medial Golgi, N-acetylglucosaminyltransferase I (GlcNAc-TI/MGAT1) introduces a GlcNAc residue to the Man5GlcNAc2 core, forming GlcNAcMan5GlcNAc2. Subsequently, through the sequential activity of enzymes such as α-mannosidase II, N-acetylglucosaminyltransferase (MGAT), and galactose-1-phosphate uridyltransferase (GALT), hybrid or complex N-glycans are synthesized. Hybrid N-glycans retain five mannose residues, while complex N-glycans lose two terminal mannoses and incorporate a second GlcNAc, forming a bi-antennary structure that can further branch and elongate.^75^ Finally, sialic acid and fucose residues are attached to the termini of complex N-glycans, increasing structural diversity and conferring additional functional properties, including the regulation of protein-protein interactions, cell-cell communication, protein stability, and ion transport.

It is noteworthy that certain bacteria, such as Campylobacter jejuni, possess N-linked glycosylation pathways. The Pgl pathway in C. jejuni facilitates the attachment of a heptasaccharide to asparagine residues within specific protein motifs. This bacterial glycosylation system has been harnessed in engineered Escherichia coli strains to produce recombinant glycoproteins, underscoring its biotechnological relevance.^76^^,^^77^^,^^78^

#### O-glycosylation pathways and their role in protein processing

**Figure 5:**
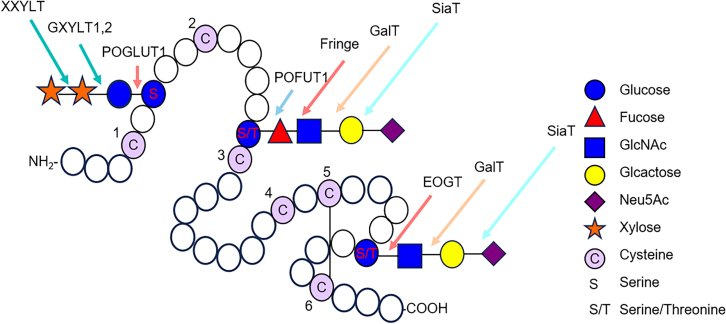
Overview of enzymes involved in O-fucosylation and O-glucosylation of specific consensus sequences on EGF repeats A single EGF repeat is shown with enzymes that act exclusively on these sequences. GlcNAc-Fuc-O can be further extended by nonspecific galactosyl- and sialyltransferases.

The Notch receptor family plays a crucial role in developmental processes, and dysregulation of its activity is implicated in numerous diseases. O-glycosylation of the epidermal growth factor (EGF) extracellular domain has been shown to modulate Notch-ligand interactions (Figure 5).^79^,^80^ The EGF domain undergoes O-fucosylation and O-glucosylation in the endoplasmic reticulum, with these initial modifications further processed in the Golgi apparatus.^38^^,^^81^ O-fucose is extended into a tetrasaccharide (SA-α2,6-Gal-β1,4-GlcNAc-β1,3-Fuc-α1-O-Ser) by the β-1-3-GlcNAc transferase of the Fringe family. Meanwhile, O-glucose is elongated into a disaccharide or a trisaccharide (Xyl-α1-3-Xyl-α1-3-Glc-β1-O-Ser) by glucoside α1-3 xylosyltransferases (GXYLT1, 2) and xyloside α1-3 xylosyltransferase (XXYLT).^82^ These glycans function as critical regulators of Notch activity, modulating interactions between the Notch receptor and its ligands, including members of the Delta and Jagged families.^83^

Beyond its role in Notch receptor regulation, O-Fucosylation is also essential for the folding and secretion of glycoproteins carrying EGF and thrombospondin type 1 repeat (TSR) domains. O-Fuc glycans stabilize these domains during maturation in the ER, preventing misfolding and facilitating efficient secretion. Recently, Hao et al. demonstrated that FUT10 and FUT11 (now named POFUT3 and POFUT4) catalyze O-Fucosylation in the EMI domain, further expanding the known biological functions of O-Fuc. They found that POFUT3 and POFUT4 localize to the ER and act specifically on pre-folded EMI domains. The O-Fuc modification by POFUT3 and POFUT4 enhances protein stability and secretion, suggesting their involvement in a non-canonical ER quality control pathway for EMI domain-containing glycoproteins.^84^^,^^85^

Furthermore, GalNAc-type O-glycosylation, a post-translational modification observed in most membrane-associated and secreted proteins, plays critical roles in diverse biological processes including embryogenesis, organ development, tissue homeostasis maintenance, and cancer progression.^86^^,^^87^^,^^88^ This modification is initiated by polypeptide N-acetylgalactosaminyltransferases (ppGalNAc-Ts), a family of up to 20 evolutionarily conserved enzymes.^89^ A functional loss study showed that ppGalNAc-T4 modulates TGF-β1 signaling by catalyzing the O-GalNAcylation of TGF-β type II receptor (TβRII) at the Ser31 site and TGF-β type I receptor (TβRI), which in turn inhibits the dimerization of TβRI and TβRII, ultimately suppressing TGF-β1 signaling and epithelial-mesenchymal transition (EMT) in human breast cancer cells.^90^ Just as reported, aberrant O-glycosylation is a hallmark of cancer, which is not only associated with the TGF-β signaling pathway but also relevant to the Wnt pathway. A study demonstrated that polypeptide N-acetylgalactosamine-transferase 1 (GALNT1), the enzyme that initiates the biosynthesis of mucin-type O-glycosylation, of which overexpression in gastric cancer activates the Wnt/β-catenin signaling pathway through the aberrant O-glycosylation of CD44, thereby promoting malignancy.^91^

#### Glycosylphosphatidylinositol (GPI) anchors in protein maturation

**Figure 6:**
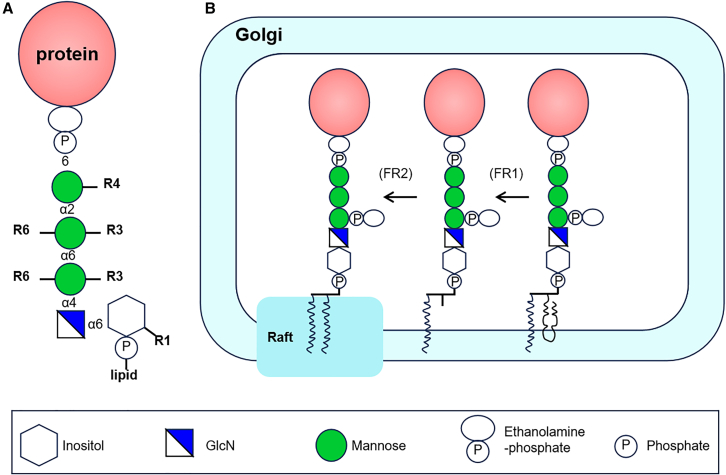
In mammalian cells, GPI-anchored protein is modified in the Golgi apparatus (A) GPI-anchored protein core structure. R1–R6, side-chains. (B) Fatty acid remodeling of GPI anchors. GPI-AP associate with membrane rafts through their lipid anchor, with fatty acid remodeling being a crucial step in this process, involving the removal of unsaturated fatty acids from the GPI anchor and their replacement with saturated fatty acids. FR, fatty acid remodeling.

Glycosylphosphatidylinositol (GPI) anchoring is a widely occurring post-translational modification in eukaryotes. GPI anchors share a conserved core structure, EtNP-6Mana1-2Mana1-6Mana1-4GlcNa1-6myoInositol-phospholipid (Figure 6A), which is found across many species. In mammalian cells, approximately 150 proteins are attached to the plasma membrane via GPI anchors.^92^,^93^ GPI anchoring secures proteins to the cell surface, imparting specific functional properties. The GPI-anchored protein undergoes fatty acid and glycan remodeling within the Golgi apparatus (Figure 6B). The glycan component of the GPI anchor plays a vital role in the transport, localization, and interactions of GPI-anchored proteins with other molecules. In yeast, as GPI-anchored proteins (GPI-APs) transit through the Golgi apparatus, a fifth mannose (Man5) is added to the fourth mannose (Man4) residue via an α1–2 or α1–3 linkage. This mannose addition is essential for the subsequent processing and proper localization of GPI-anchored proteins.^38^^,^^93^^,^^94^ In parasites such as *Trypanosoma brucei*, the glycan component of GPI-anchored proteins undergoes further specialized modifications. In the procyclic form of *T. brucei*, a β1-3-linked GlcNAc is added to the second galactose (Gal) residue on the GPI anchor by GlcNAc transferase. Subsequently, trans-sialidase transfers sialic acid from a sialic acid sugar conjugate to the GPI-anchored side chain.^95^

In summary, glycosylation plays an indispensable role in protein folding and maturation. Beyond functioning as a quality control mechanism to ensure proper folding and stability, glycosylation actively promotes protein maturation and modulates biological activity by regulating protein function.^96^

### Thermodynamic mechanisms by which glycosylation modulates *in vitro* protein folding

Glycans are hydrophilic biomolecules formed by the linkage of monosaccharides through glycosidic bonds, which can significantly alter protein properties,^97^ including bioactivity, chemical solubility, folding, and stability.^98^^,^^99^^,^^100^ Glycosylation has been demonstrated to enhance the thermal stability of proteins and accelerate folding kinetics,^101^^,^^102^ thereby exerting a positive effect on protein folding *in vitro*. Specifically, N-linked glycosylation stabilizes folding intermediates, reduces aggregation, and improves the folding efficiency of glycoproteins by limiting structural flexibility and increasing thermodynamic stability.^103^ However, the thermodynamic principles behind this understanding, including enthalpic and entropic factors, have been the subject of early debates.

#### Enthalpic contributions of glycosylation to protein folding stability

Glycosylation significantly influences protein stability through enthalpic interactions. The covalent attachment of glycans to specific asparagine residues (N-linked glycosylation) on the protein surface can further enhance the thermal and kinetic stability of the protein to stabilize the folded state. This stabilization is primarily enthalpic, favorable glycan–protein interactions (e.g., hydrogen bonding, van der Waals forces) contribute enthalpic stabilization by lowering the free energy of the folded state.^104^^,^^105^^,^^106^

**Figure 7:**
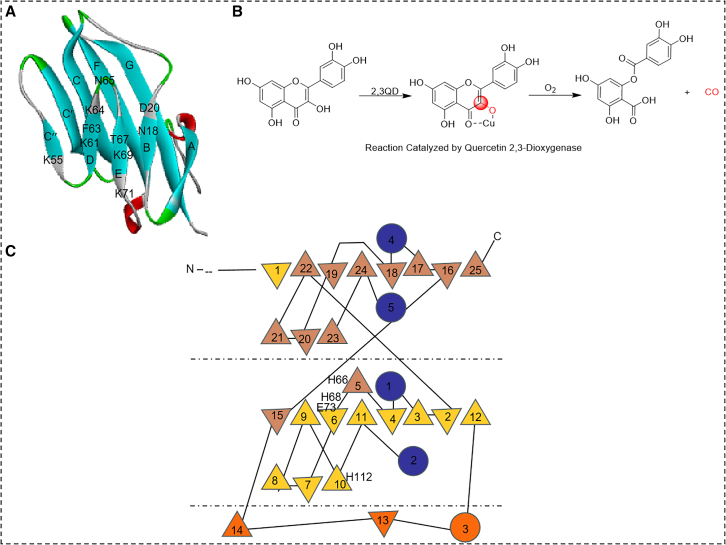
Enthalpic contributions of glycosylation to protein folding stability (A) Ribbon diagram of a single representative CD2 structure consisting of two p-lamellae containing chains D, E, B, C", C', C, F, and G. CD58 binding sites include residues in the C, C', and F chains, as well as CC", C'C", and FG' rings, located in glycoproteins. Lys55, Lys64, and Lys71 together with Lys69 form clusters of positively charged residues around Lys61. (B) Reaction catalyzed by quercetin 2,3-dioxygenase. (C) Topology diagram of the secondary structure of the 2,3QD monomer.

Structural studies have provided insights into these stabilizing interactions. For instance, Wagner et al. resolved the structure of a high-mannose N-glycan [-GlcNAc2-Man5-8] in the adhesion domain of human CD2 and found that the N-glycan does not participate in protein-protein interactions between CD2 and other protein^107^. Instead, it stabilizes protein folding by counterbalancing unfavorable aggregation caused by five positive charges centered around Lys61 in CD2 (A of Figure 7). Similarly, Dijkstra’s study^108^ on the crystal structure of copper-containing quercetin 2,3-dioxygenase from Aspergillus japonicus revealed that N-linked glycosylation sites are involved in stabilizing the protein structure through specific interactions at the domain interface (B and C of Figure 7).

Furthermore, glycosylation can influence the folding pathway of proteins by modulating the energy landscape. Because the large-volume amphiphilic modification on asparagine can intrinsically enhance solubility and folding energy through carbohydrate-protein interactions. N-linked glycans can also externally enhance glycoprotein folding by utilizing the glycoprotein homeostasis or "glycoprotein homeostasis" network.^109^ This enthalpic contribution is crucial in ensuring proper protein folding and stability, particularly in the cellular environment where misfolding can lead to aggregation and loss of function.^110^

Molecular dynamics simulations have provided significant insights into how glycosylation modulates the enthalpic contributions during protein folding. For instance, MD studies have demonstrated that N-glycosylation can enhance hydrogen bonding networks and van der Waals interactions, leading to increased stability of the folded state. These simulations reveal that glycan moieties can form specific interactions with protein residues, thereby lowering the enthalpic component of the folding free energy and promoting a more stable native conformation.^111^^,^^112^

In summary, glycosylation maintains the native conformation of proteins by enhancing their stability through affinity mechanisms, reducing unfavorable solvent interactions, and modulating folding pathways to form a stable folding state.

#### Entropic modifications by glycosylation in protein folding efficiency

Glycosylation significantly influences protein folding and stability through entropic effects.^113^ The covalent attachment of glycans restricts the conformational space available to the unfolded protein, reducing its entropy and making the unfolded state less thermodynamically favorable. This entropic restriction promotes the transition to and stabilization of the folded state, enhancing folding efficiency and stability. For instance, the Kawasaki group demonstrated that N-glycosylation plays a critical role in preventing protein degradation and instability, further underscoring its importance in maintaining protein functionality.^114^

**Figure 8:**
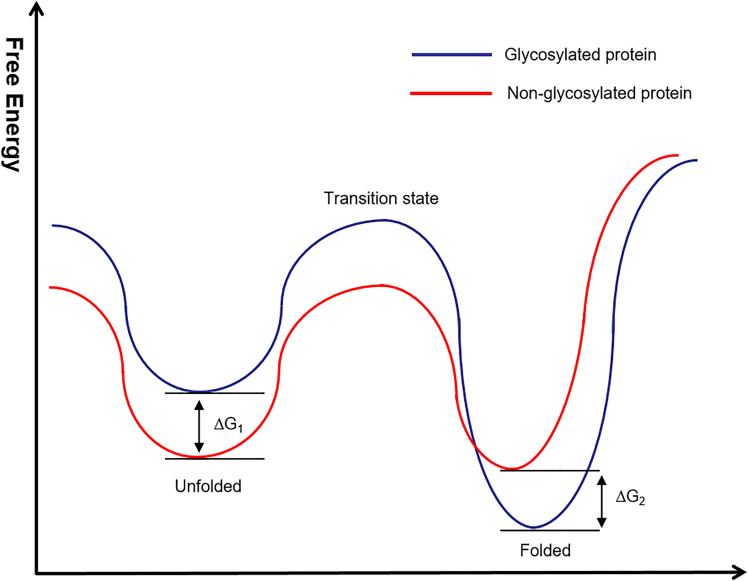
The difference in free energy between glycosylated and non-glycosylated proteins in the folded and unfolded states

Levy conducted a systematic thermodynamic analysis *in vitro* (Figure 8), showing that glycosylation does not stabilize the folded state directly but instead destabilizes the unfolded state. Glycans impose constraints on the unfolded state, limiting the formation of residual native structures and increasing their free energy. This effect shifts the folding equilibrium toward the native conformation. Interestingly, Levy also observed in the study that when more glycans were added, there was a slight increase in entropy in the unfolded state.^115^

Furthermore, glycan position and size play critical roles in determining protein stability. Glycosylation at specific sites can modulate folding pathways by imposing structural constraints, which can either facilitate or hinder proper folding.^116^ Despite these insights, detailed molecular-level mechanisms remain elusive due to earlier challenges in preparing homogeneously glycosylated proteins with site-specific modifications. Advances in chemical and chemoenzymatic methods are expected to address these gaps and enable a deeper understanding of glycosylation’s role in protein folding stability.

In summary, glycosylation enhances protein folding and stability by modulating the entropy of the unfolded state. The ability of glycans to influence protein conformations, prevent aggregation, and stabilize folding pathways highlights their crucial role in protein biophysics and the development of glycoprotein-based therapeutics.

### Influence of N-glycosylation on protein folding kinetics and stability

#### Structural and stability effects of N-GlcNAc2 on protein folding

**Figure 9:**
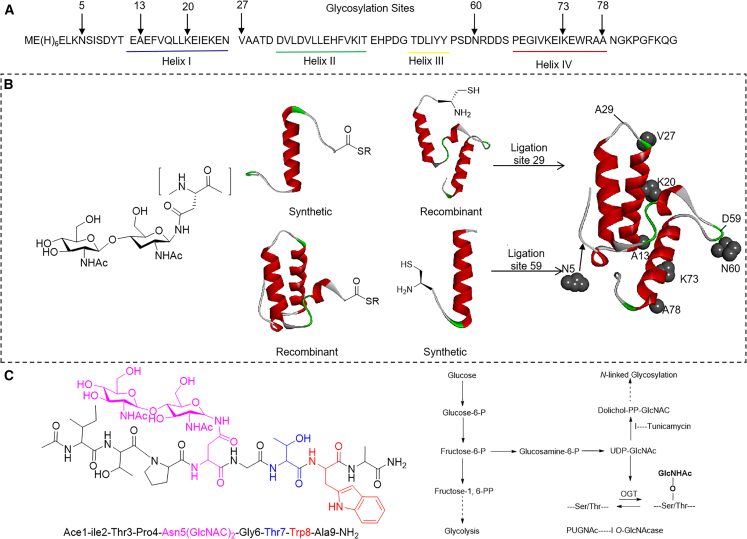
The effect of N-GlcNAc2 on protein secondary structure (A) Amino acid sequence of amino-Im7 shows helices I–IV and 7 glycosylation sites. (B) On the left is the structure of chitobiose-ASN, and on the right is the Band diagram of Im7, showing positions 29 and 59 that mutate to cysteine, thus enabling a linking strategy for the introduction of glycosyl ASN. The native Im7 residues on each selected glycosylation are also shown in the space fill plot (black). (C) On the left is the chemical structure and sequence of glycopeptide, and on the right is the hexosamine biosynthetic pathway and O-GlcNAc glycosylation.

Imperiali^117^ used bacterial immunity protein Im7 as a model to explore the effects of glycosylation on protein folding (A and B of Figure 9). The study found that the disaccharide of the GlcNAc-GlcNAc structure disrupts the α-helix, but glycosylation at the terminus of the α-helix does not affect the structure, and the size and conformation of the glycan also have an impact on protein stability and structure.

Noid’s^118^ molecular dynamics simulations demonstrated that the disaccharide structure of glycosylated proteins has a smaller impact on the solvation layer compared to the peptide backbone itself. However, excessively large glycans introduced during N-glycosylation can hinder the collapse of hydrophobic regions, a critical step in protein folding, potentially leading to folding failure. Separately, the Isono group^119^ revealed that increased glycosylation under glucose-deprivation conditions, detected by the CTD110.6 antibody, is due to N-GlcNAc2 modifications rather than O-GlcNAc. Unlike conventional O-GlcNAcylation, which is regulated by OGT and O-GlcNAcase, this induced glycosylation is inhibited by the N-glycosylation inhibitor tunicamycin, highlighting distinct regulatory mechanisms for these modifications (C of Figure 9).

Computational studies, particularly molecular dynamics simulations, have elucidated the impact of N-GlcNAc2 glycosylation on protein secondary structure. These simulations indicate that the attachment of N-GlcNAc2 can induce conformational changes that stabilize α-helices and β-sheets, depending on the glycosylation site. For example, MD simulations have shown that glycosylation at specific asparagine residues can lead to localized structural rigidity, thereby influencing the overall folding pathway and stability of the protein.^120^

#### Preferred sequence motifs for N-glycan-mediated protein folding

N-linked glycosylation does not occur at every potential glycosylation site, even between different molecules of the same protein. Additionally, N-glycans have a specific influence on the sequence position of protein folding. Kelly^121^ explored the principles of the N-glycan promotion of protein folding and stability *in vitro*, combining experimental and computational methods, and found that when an aromatic sequence in the turn structure is added to N-glycans, the Asn residue is usually located at the turn. This structure stabilizes the native interactions between Phe, Asn-GlcNAc, and Thr, reducing the equilibrium concentration of misfolded or unfolded proteins, thereby promoting glycosylated protein folding.

#### N-glycosylation facilitates the folding of large proteins (>300 amino acids) *in vitro*

**Figure 10:**
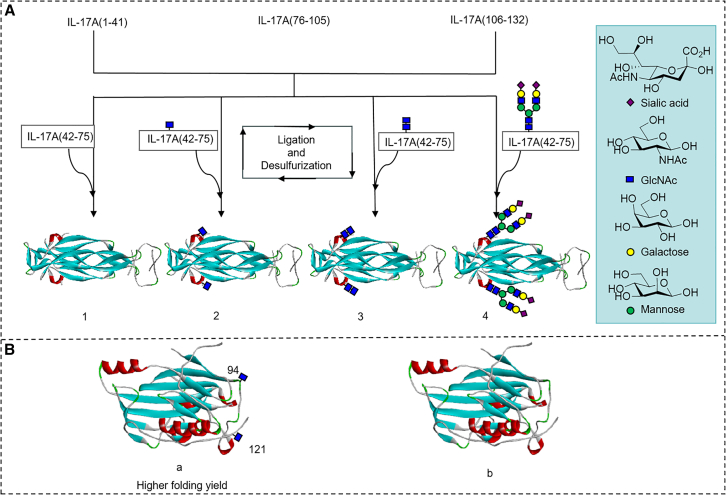
N-glycans promote the folding of large proteins (over 300 amino acids) *in vitro* (A) General strategy for synthesizing several (glyco) isoforms of IL-17A. (B) The folding rate of synthesized GlcNAc containing gD was higher than that of unglycosylated gD.

Recently, Dong^122^ investigated the impact of N-glycosylation on the stability of protein folding *in vitro* (A of Figure 10). The study found that for IL-17A, glycosylated proteins fold into homodimers more easily than native proteins without glycosylation. Furthermore, larger glycan chains convert oxidized intermediates into monomers faster than monosaccharides, subsequently folding into dimers at a higher yield. Wang^123^ explored the structure-activity relationship of glycan types in receptor-binding protein glycoprotein D (gD) of herpes simplex virus 1 (HSV-1). They found that glycosylated gD synthesized with different homogeneous glycans had a higher folding yield than non-glycosylated gD (B of Figure 10).

Molecular dynamics simulations have been instrumental in understanding how N-glycans facilitate the folding of large proteins exceeding 300 amino acids. These computational studies suggest that N-glycans can act as molecular chaperones, preventing non-specific aggregation by shielding hydrophobic regions during the folding process. Additionally, MD simulations have revealed that glycans can modulate the energy landscape of protein folding, reducing the likelihood of misfolded intermediates and promoting efficient attainment of the native state.^112^

### O-glycosylation and its impact on protein folding efficiency *in vitro*

O-glycosylation is crucial for the physiological function of proteins and plays an essential role in regulating cell growth and the cell cycle during early developmental stages. It is a mechanism for regulating protein-protein interactions and intercellular signal transduction. The most common type of O-glycosylation is the addition of GalNAc to the hydroxyl group of serine or threonine, forming the core structure typically referred to as mucin-type glycosylation.^124^ Previous reports have shown that O-glycosylation is also related to protein folding *in vivo*.^37^ How O-glycosylation participates in protein folding and quality control is still unclear, and it remains unknown whether it affects other closely related folding characteristics or which structural features determine these effects.

**Figure 11:**
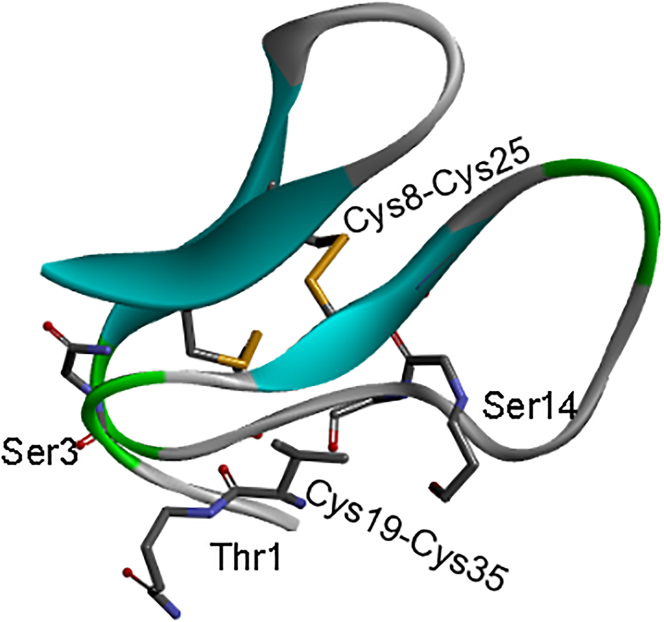
Structure of CBM with three O-linked monomannose residues at Thr1, Ser3, and Ser14

Tan^125^ used precise chemical synthesis of homogeneously O-Man glycosylated CBM proteins (carbohydrate-binding modules from Trichoderma reesei cellulase TrCel7A) to quantify, for the first time, the contribution of O-glycosylation to oxidative protein folding. CBM has three O-Man residues at the Thr1, Ser3, and Ser14 sites (Figure 11). The results showed that O-glycosylation can accelerate the protein folding rate, and the effect is more pronounced when glycosylation occurs at specific sites such as the *N*-terminus. The impact of multiple O-glycosylation modifications on the folding rate depends on their positions and the structural flexibility of the protein. Since O-Man is added in the ER and subsequently diversified in the Golgi apparatus *in vivo*, it is speculated that the addition of single mannose in the ER may function similarly to N-glycans, essentially to accelerate proper protein folding *in vivo*.

## The role of glycosylation in protein folding *in vivo* and *in vitro*

Few have linked the effects of glycans on protein folding stability *in vitro* to their functions *in vivo*. This is because obtaining glycoproteins with homogeneous glycan structures remains a technical challenge for most laboratories. Advances in protein chemical synthesis have made the production of homogeneous N-glycoproteins possible, allowing some research groups to use them as biological probes to elucidate the functions of different types of glycans *in vivo*.

### Synthetic glycoprotein probes for investigating the glycoprotein quality control (GQC) cycle

The glycoprotein quality control (GQC) system in the ER is crucial for producing both correctly and incorrectly folded glycoproteins. In the GQC system, UGGT plays a central role in distinguishing between correctly and incorrectly folded protein structures.^126^

**Figure 12:**
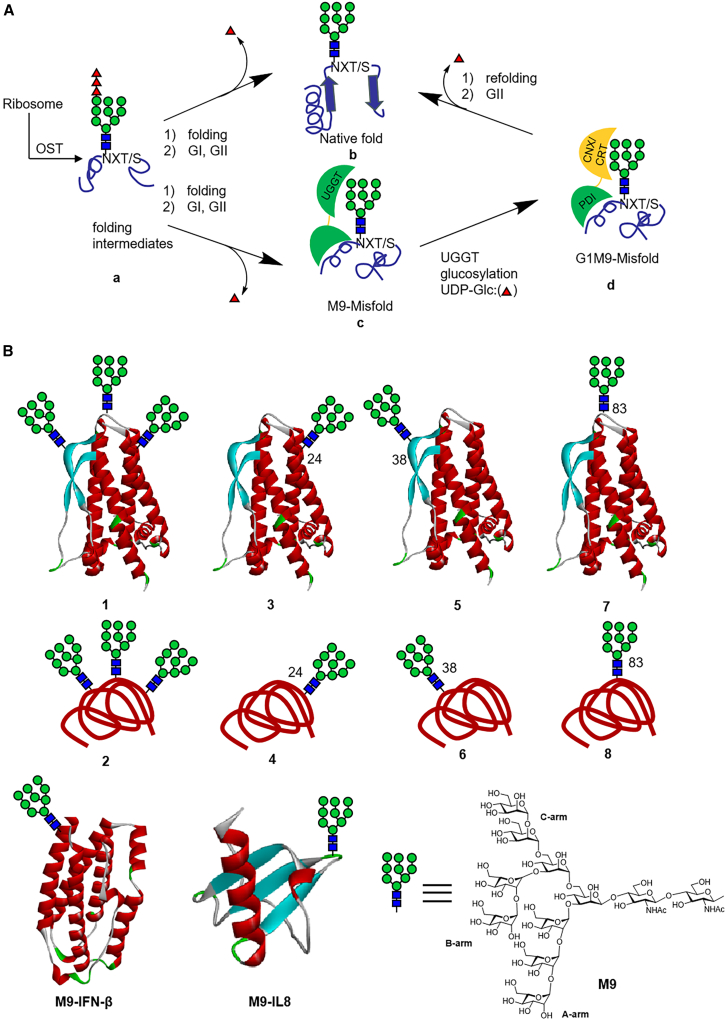
Glycoprotein quality control (GQC) systems and chemical synthesis of glycoproteins (A) Polypeptides synthesized by ribosomes undergo glycosylation under OST cotranslation, and glycosylated polypeptides with G3M9-glycan begin the folding process. Misfolded glycoproteins are refolded by the CNX/CRT cycle. (B) The structure of chemically synthesized homogeneous glycoprotein, erythropoietin (EPO), with a homogeneous M9 hypermannose-type glycan at positions 24, 38, and 83 (3, 5, 7), and with three glycans1. The misfolded EPO contained three glycan2, and also one M9 hypermannose-type glycan at positions 24, 38, and 83 (4, 6, 8). At the bottom are the structures of chemically synthesized interferon (IFN-β) and interleukin (IL)-8.

Building on previous research, Kajihara^127^ chemically synthesized a series of homogeneous glycoproteins with M9-high-mannose oligosaccharides, including erythropoietin (EPO), interferon-β (IFN-β), and interleukin-8 (IL-8), as well as their monomeric, dimeric, and aggregated misfolded forms. These were used to study the molecular mechanisms behind the glycoprotein quality control (GQC) system (Figure 12).

*In vitro* experiments revealed that UGGT (uridine diphosphate glucose glycoprotein glucosyltransferase) transfers glucose residues to all native and misfolded M9-EPO without site specificity. Surprisingly, UGGT was unable to distinguish between the native and misfolded forms of EPO, suggesting it mistakenly recognizes native EPO as a misfolded protein. However, UGGT exhibited a stronger ability to distinguish other glycoproteins, as it only transferred glucose to the misfolded forms of M9-IL-8 and M9-IFN-β. This finding highlights structural differences between EPO and other glycoproteins, likely originating from the hydrophobic surface regions of misfolded proteins. Although M9-glycans are hydrophilic, EPO has a large hydrophobic surface area, likely exposing more hydrophobic patches than IL-8 and IFN-β. This hydrophobic exposure may serve as a signal for UGGT recognition, facilitating glucose transfer to misfolded proteins.

In ER lysate experiments, UGGT did not glycosylate either the native or misfolded forms of EPO, indicating that the interaction between UGGT and glycoproteins is more complex *in vivo* than previously thought. This discrepancy prompted them to investigate the crosstalk between glycosylation and de-glycosylation events in the ER. Using isotope tracking and GI inhibition, they demonstrated that M9-EPO and G1M9-EPO undergo rapid glycosylation/de-glycosylation cycles, which are difficult to observe *in vitro*. These findings suggest the presence of a dynamic, highly regulated GQC system that balances protein folding and glycosylation in real time.

Further investigation into the folding ability of misfolded glycoproteins revealed that while monomeric and dimeric misfolded M9-IL-8 could refold into their native conformations in ER lysates, aggregated misfolded M9-EPO could not. This suggests that some misfolded glycoproteins, particularly those with more complex structures, may escape the GQC system and be targeted for degradation instead. The inability to refold aggregated misfolded M9-EPO highlights the structural limitations imposed by glycan-mediated folding.

Furthermore, misfolded G1M9-IL8 was successfully refolded into its native M9-IL-8 form in ER lysates, further demonstrating the rapid glycosylation/deglycosylation process of the GQC system. This finding highlights the overall, this study highlights the complex interplay between glycans and protein folding, where hydrophobicity, glycosylation, and the GQC mechanism coordinate to maintain protein homeostasis. By utilizing chemically synthesized homogeneous glycoproteins, this study revealed the molecular details of the GQC cycle, demonstrating the utility of glycoprotein probes in investigating the roles of different glycans *in vivo*.

### Chemical synthesis of homogeneous glycoproteins for live-cell studies of N-Glycan maturation

The research groups of Kajihara and Sato^128^ designed and chemically synthesized an intermediate N-glycan structure—cholera toxin B subunit (CTB) modified with a complex-type heptasaccharide. The synthesized glycoprotein was successfully monitored as being transported to the ER and Golgi apparatus in living cells. Further, different glycan-modified CTBs were prepared to study the function of N-glycans in cells and the structural changes of N-glycans after incubation in living cells. This indicates that synthesized CTB can be used for delivering and studying the function of homogeneous N-glycans in specific organelles of living cells.

### Alternative production systems for glycoproteins

#### Engineered bacteria (C. Jejuni)

Engineered C. jejuni strains are widely used in scientific research because of the advantages of a simple system, fast cycle, and low cost of the experiment. It was the first bacterium to be identified as having a “universal glycosylation system” that modifies proteins extensively.^129^ In Campylobacter jejuni, N-glycosylation affects bacterial adhesion and invasion *in vitro*, as well as the recognition of innate and adaptive immune responses.^130^ Moreover, N-connexin glycosylation is a necessary condition for Campylobacter jejuni to fully function.^131^ The biosynthesis and transfer of N-glycan to protein involves the action of 10 Pgl proteins and begins with the synthesis of nucleotide-activated UDP-diNAcBac from UDP-GlcNAc in the cytoplasm, which is catalyzed by the activities of PglF dehydratase (rate-limiting enzyme), PglF aminotransferase, and PglD acetyltransferase.^129^ In the study of relevant mechanisms, the N-glycosylation pathway of Campylobacter jejuni involves oligosaccharide transferase (OST) PglB transferring a conserved heptasaccharide N-glycan to the asparagine residues of the extended sequence fragment D/E-X1-NX2-S/T (where X1 and X2 are any amino acids other than proline) on the neointimal side of the neoplasma.^132^^,^^133^^,^^134^ They are therefore limited in the types of glycan structures they can synthesize.

#### Yeast systems

Yeast is a valuable host for the production of recombinant proteins, with the main advantages of stable production strains, durability, the possibility of high-density growth, high yield and productivity, and the ability to perform post-translational modification.^135^^,^^136^^,^^137^ In the existing research, Main hosts using yeast for glycoprotein production include Saccharomyces cerevisiae, Komagataella pastoris (P. pastoris), Hansenula polymorpha, Kluyveromyces lactis, and Yarrowia lipolytica.^135^^,^^138^^,^^139^^,^^140^^,^^141^^,^^142^ Among them, Saccharomyces cerevisiae has high safety and has been confirmed as a safe organism by the FDA, and is a good substitute for E. coli. As the second generation yeast expression system, Komagataella pastoris has easier genetic operation, higher protein expression level, and stronger protein modification ability. One of the advantages of Kluyveromyces lactis as a host over Saccharomyces cerevisiae is that it metabolizes hexose through glycolysis and pentose phosphate pathways. Hansenula polymorpha is a methylotrophic yeast that can grow with methanol as its only carbon and energy source.^141^ Yarrowia lipolytica has the potential to produce lipases and proteases.^139^

Yeast glycosylation has many advantages, such as scalability,^143^^,^^144^^,^^145^^,^^146^ as well as limitations, such as supermannosylation.^147^ A key difference between yeast and mammalian cells is the type of glycosylation structure, with supermannosylated glycosylation structures that trigger an immune response.^148^ For pathogen-host interactions, both pathogenicity and host resistance (susceptibility) are attributed to glycosylation.^149^ Modifying a protein by altering the glycosylation structure has both advantages and disadvantages for the infected organism, as it affects host resistance and pathogen virulence.

#### Insect cells

Insect cells have post-translational modification, processing, and sequencing mechanisms similar to mammalian cells, which facilitate the production of correctly folded proteins.^150^^,^^151^ In addition, the transient gene expression system is one of the most important techniques for protein function analysis in the *in vitro* cell culture system of Bacillus viruses, which was developed to express foreign genes in the transient expression vector under the control of insect virus promoters.^152^ Transient insect cell expression provides the possibility for rapid synthesis of recombinant proteins.

Glycosylation is an important property of insect cell expression systems, but some insect cell lines produce core alpha 1,3-fucosylated N-glycans, which are highly immunogenic, making recombinant glycoproteins unsuitable for human use. Jarvis et al.^153^ therefore constructed a rod-virus vector designed to express RMD immediately after infection and facilitate the insertion of genes encoding any glycoproteins of interest for later expression after infection. The results show that our method can be used to solve the problem of immunogenic core α1,3-fucosylation associated with insect cell systems. Fatemeh et al.^154^ developed a gene expression system that uses recombinant baculoviruses to express full-length coding sequences under multiparty promoters in SF-9 cells. Recombinant Gn was purified by affinity chromatography, and the immunoreactivity of the protein was assessed using serum from patients with confirmed CHF infection. The results showed that the recombinant En protein was highly immunogenic and could induce high titer antigen-specific antibodies. Induction of the inflammatory cytokine interferon-gamma and the regulatory cytokine IL-10 was also detected.

Insect cells have gradually entered the field of researchers because insect cells do not carry any human infectious viruses, have efficient replication, and low cost.^155^^,^^156^ However, proteins synthesized by insect cells differ in modification from mammalian proteins, and differences in glycosylation modification affect immunogenicity, and these properties may be related to differential effector function and cytokine signaling.^157^

#### Mammalian cells

Chinese hamster ovary (CHO) cells are the most commonly used mammalian host cell system in the industrial production of biological products. Its main applications for heterologous recombinant protein expression lie in the relative simplicity of the stable introduction of ectopic DNA into the CHO host cell genome and its suitability for growth in suspension culture.^158^^,^^159^ Additionally, HEK293 cells, baby hamster kidney (BHK) cells, NS0 myelom, Sp2/0 hybridoma mouse cell lines and HT-1080 human cells are also commonly used mammalian host cell systems.^160^^,^^161^^,^^162^ These cell lines are capable of producing human-like glycosylation patterns, making them ideal for therapeutic glycoproteins, but also with high costs and restriction condition.^158^

### Glycosylation in therapeutic proteins: Enhancing folding *in vitro* and extending serum stability

**Figure 13:**
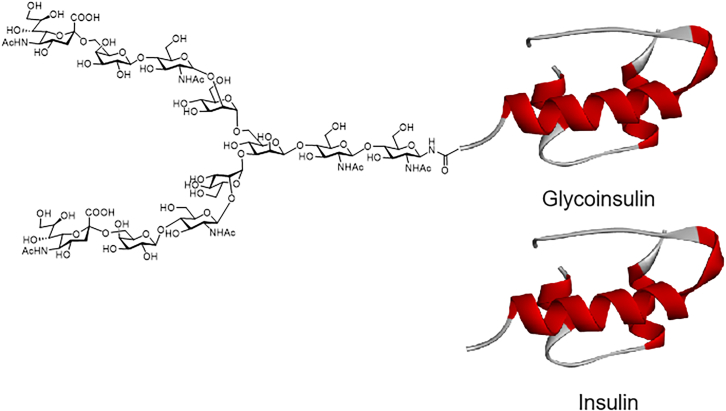
N-glycosylation inhibits the formation of insulin fibrils Insulin with or without N-glycosylation modification.

The research groups of Hossain and Wade^163^ reported the chemical synthesis of human disialo-glycoinsulin on the B-chain (Figure 13). Although introducing large glycans at certain positions might affect protein folding, experimental validation showed that disialylation did not impact insulin’s structure. *In vivo* experiments demonstrated that glycosylated insulin retained its natural binding affinity to insulin receptors while enhancing its stability in serum.

## Nature-inspired glycosylation strategies for enhancing protein folding

In natural systems, glycosylation helps protect proteins from aggregation, promotes proper folding, and shields them from degradation. Inspired by these natural processes, Shi and co-workers have developed a glycosylation-based method that assists in the chemical synthesis and correct folding of complex proteins, especially those with challenging folding requirements, such as disulfide-bond proteins.

### Removable O-linked β-N-acetylglucosamine as a transient folding scaffold

**Figure 14:**
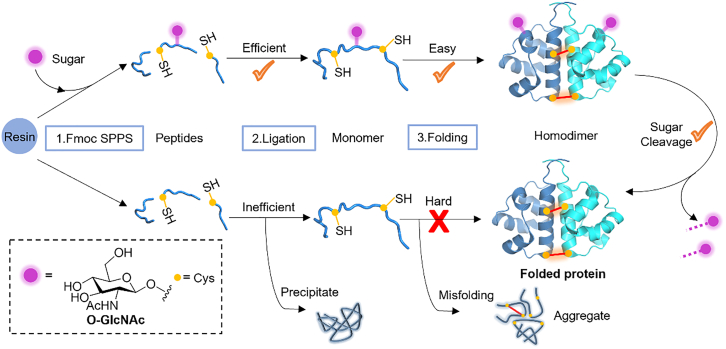
Removable O-linked β-N-acetylglucosamine promotes the efficient folding of chemically synthesized proteins

In 2022, Shi and co-workers introduced a strategy employing removable O-linked β-N-acetylglucosamine (O-GlcNAc) as a folding scaffold during the chemical synthesis of disulfide-rich proteins.^33^ This sugar temporarily attaches to the protein, helping it folds properly by stabilizing key parts of the molecule. Once the protein is folded, the sugar can be removed by glycosidase O-GlcNAcase, leaving the protein in its natural form without any changes to its structure (Figure 14). Using this method, two proteins were successfully synthesized and folded: Hepcidin, a small disulfide-rich peptide hormone, and interleukin-5 (IL-5), a homodimeric cytokine.

Hepcidin, which contains multiple disulfide bonds, is particularly challenging to fold due to its tendency to misfold or form incorrect disulfide bridges. The RGM strategy helped guide the proper formation of these bonds, resulting in a correctly folded and functional protein. In the case of IL-5, the RGM strategy played a crucial role in ensuring that the two identical subunits of the homodimer folded and assembled into the correct structure, which is essential for its biological activity.

This approach mirrors how glycosylation works in nature by temporarily stabilizing the protein during its folding process. Once the protein is fully folded, the sugar modification can be removed, leaving the native protein structure intact. This strategy is particularly effective in managing the folding of disulfide-rich proteins, where controlling bond formation is key to obtaining a functional protein.

### L-glycosidase-cleavable glycans for facilitating native folding of D-proteins

**Figure 15:**
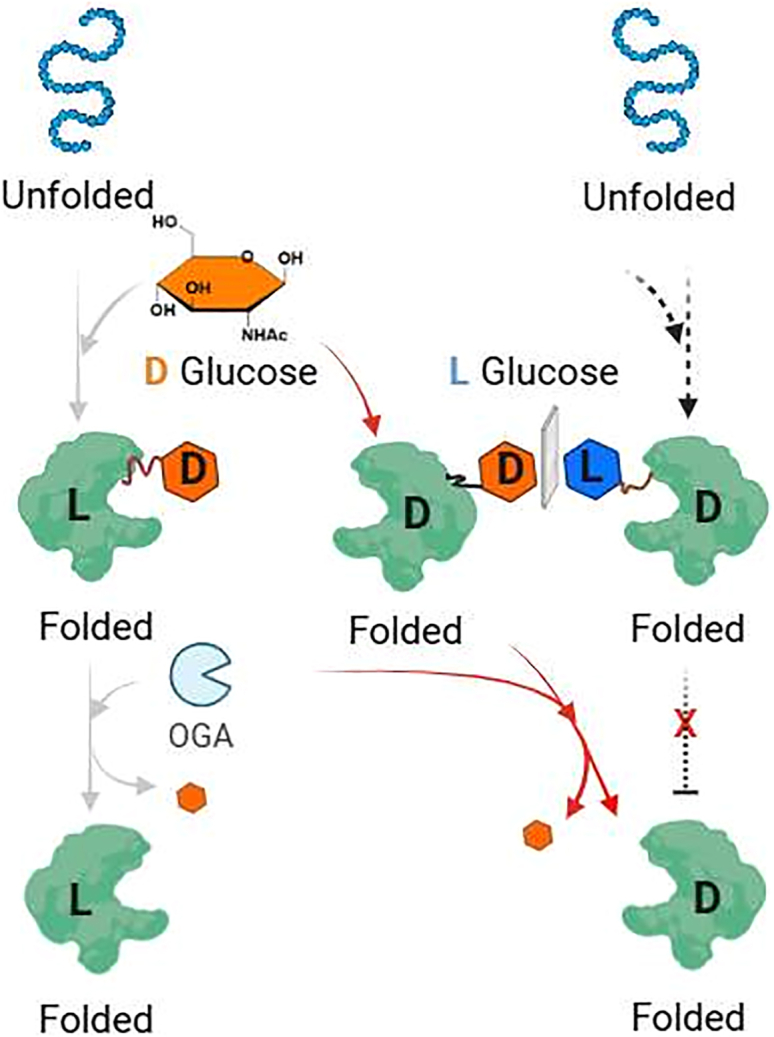
Natural sugars promote the folding of D-proteins

Additionally, Shi explored the use of L-glycosidase-cleavable natural glycans to assist in the folding of chemically synthesized mirror D-proteins (Figure 15).^47^ D-proteins, which are synthetic counterparts of natural proteins with reversed chirality, hold great potential for mirror-image drug screening, as they are resistant to degradation by natural enzymes.^11^^,^^28^^,^^164^ A few mirror D-proteins were successfully synthesized and folded, such as D-RBD, a mirror image of the receptor-binding domain of the SARS-CoV-2 spike protein; D-TNFa, a mirror form of the tumor necrosis factor-alpha, and D-Trka^48^; a mirror form of the nerve growth factor receptor.

Efficient folding of these mirror D-proteins was achieved by utilizing natural sugars and enzymes. The glycan modification stabilized the folding intermediates, preventing aggregation and incorrect folding, which are common challenges in the synthesis of such proteins. After folding was complete, the glycans were selectively removed, yielding correctly folded, functional mirror proteins. These proteins are particularly valuable for “Mirror screening,” which identifies mirror ligands that bind tightly to these proteins and resist enzymatic degradation.

The use of glycosylation in chemical protein synthesis provides an effective way to guide the correct folding of complex proteins. While challenges remain in fully replicating the precise control of glycosylation seen in living systems, methods such as the RGM strategy and L-glycosidase-cleavable glycan modification offer powerful tools for ensuring correct protein folding. High-quality proteins with the correct structures, including disulfide-rich proteins and mirror proteins for advanced applications such as drug screening, can be produced by mimicking nature’s methods.

## Conclusion and perspective

Glycosylation plays an essential role in protein folding, stability, and maturation within biological systems. In natural settings, glycosylation facilitates protein folding by stabilizing intermediates, preventing aggregation, and supporting quality control mechanisms within the endoplasmic reticulum (ER). However, for chemically synthesized proteins, the absence of these natural folding pathways presents considerable challenges, particularly when dealing with complex proteins containing multiple disulfide bonds or large tertiary structures. These limitations frequently lead to misfolding and reduced yields.

Previous studies on glycosylation in protein folding have typically focused on “permanent glycosylation,” where glycosylation continues to influence protein function after folding is complete, serving as a key determinant of protein stability, localization, and activity. While these findings have enhanced our understanding of glycosylation’s biological role, they do not address the specific challenges encountered in chemical protein synthesis, where folding control must be both precise and reversible. Our temporary glycosylation scaffold strategy overcomes these challenges by introducing reversible glycosylation steps during chemical synthesis. Rather than functioning as a final structural element, glycosylation is employed as a temporary scaffold to stabilize folding intermediates and facilitate the attainment of the correct conformation. After folding is complete, the glycan scaffold is removed, leaving a correctly folded functional protein devoid of residual glycosylation. This approach has proven effective in the synthesis and folding of complex proteins, including mirror proteins.

Although the use of glycosylation as a temporary scaffold has demonstrated considerable potential, further research is required to optimize this technique for a broader range of proteins. Future studies could focus on refining the structure and size of glycan scaffolds to enhance their efficacy in guiding the folding of diverse proteins. Additionally, combining glycosylation scaffolds with other folding aids, such as chemical chaperones or redox agents, may provide improved control over the folding process in *in vitro* synthesis environments. Overall, our strategy represents a significant step in overcoming the folding challenges associated with synthetic proteins and opens up new opportunities for the production of functional, high-quality proteins for therapeutic and biotechnological applications.

## Data and code availability

No data was used for the research described in the article.

## Acknowledgments

This work was supported by 10.13039/501100001809National Natural Science Foundation of China (No. 22307061), the 10.13039/501100002858China Postdoctoral Science Foundation (No. 2022M721801), and the National Key Research and Development Project of China (2024YFA0919302).

## Author contributions

**C.H.**: Writing - original draft, visualization, investigation, and conceptualization. **Q.Z.**: Writing - original draft, visualization, investigation, and conceptualization. **X.B.**: writing - review and editing and conceptualization. **W.S.**: writing - original draft, review and editing, supervision, investigation, and funding acquisition.

## Declaration of interests

The authors declare that they have no known competing financial interests or personal relationships that could have appeared to influence the work reported in this article.
